# Sample-Wise Aiding in GPS/INS Ultra-Tight Integration for High-Dynamic, High-Precision Tracking

**DOI:** 10.3390/s16040519

**Published:** 2016-04-11

**Authors:** Yanhong Kou, Han Zhang

**Affiliations:** School of Electronic and Information Engineering, Beihang University (BUAA), Beijing 100191, China; zhan1896@purdue.edu

**Keywords:** ultra-tight coupling, GPS/INS integration, Doppler aiding, high dynamics, tracking loop

## Abstract

By aiding GPS receiver tracking loops with INS estimates of signal dynamics, GPS/INS ultra-tight coupling can improve the navigation performance in challenging environments. Traditionally the INS data are injected into the loops once every loop update interval, which limits the levels of dynamics accommodated. This paper presents a sample-wise aiding method, which interpolates the aiding Doppler into each digital sample of the local signal to further eliminate the dynamic errors. The relationship between the tracking error and the aiding rate is derived analytically. Moreover, the effects of sample-wise aiding using linear and spline interpolations are simulated and compared with traditional aiding under different INS data update rates. Finally, extensive tests based on a digital IF (intermediate frequency) signal simulator and a software receiver validate the theoretical equations and demonstrate that the dynamic stress error can be significantly reduced by sample-wise aiding.

## 1. Introduction

GPS (global positioning system) and INS (inertial navigation system) have complementary characteristics and their integration has been widely explored [1]. High dynamics and high precision impose conflicting requirements on noise bandwidths of conventional tracking loops in GPS receivers [2]. GPS/INS ultra-tight coupling or deep integration can relieve this conflict by aiding the tracking loops with inertial measurements and, thus, boost immunity to high dynamics, severe interferences and brief line-of-sight blockages [3,4,5]. The idea of using ultra-tight GPS/INS integration to improve performance in challenging environments has drawn wide attention of the navigation community [6,7,8,9,10,11,12,13]. Yang, Y. & El-Sheimy, N. discussed the implementation and performance analysis of a PLL (phase locked loop) aided by a MEMS (micro electromechanical system) IMU (inertial measurement unit) [10]. Details of the IMU mechanization and state propagation can be found in [8]. Alban, S., *et al*., used a feed-forward aided PLL model and its transfer function to facilitate the performance analysis of carrier tracking loops with external aiding [11]. Gebre-Egziabher, D., *et al*., quantified the effects of tracking loop bandwidth, receiver clock jitter, aiding Doppler error and platform vibration on the quality of the carrier phase estimate provided by the aided PLL [12]. Chiou, T.Y. took account of different orders of carrier tracking loops with external aiding and detailed the process of data collection and experimental setup [13]. With the development of vector tracking based receiver techniques [14], the GPS/INS deep coupling architecture (where the tracking loops are implemented as part of a larger navigation filter) has been studied increasingly by a number of authors. Gustafson, D., *et al*., proposed a vector-based GPS receiver which can be deeply integrated with inertial and alternate sensors [15]. Petovello, M.G. & Lachapelle, G. investigated three different Kalman filter implementation options in both scalar-tracking mode and vector-tracking mode with particular consideration for carrier phase tracking [16]. The analyses and tests in [17,18] showed improved tracking and reacquisition performances of vector-based deep integration at the cost of increased computational loads. Babu, R. & Wang, J. noticed that, typically, the integration filter runs at a rate of 1 to 100 Hz, while the carrier tracking loop normally runs at about 1000 Hz [19]. An interpolator based on a CIC (cascaded integrator comb) filter is proposed to interpolate the low rate Doppler measurements from the navigation filter to 1000 Hz. Babu, R. & Wang, J. concerned the quality of IMU-derived Doppler estimates for integration, and derived the relationship between the baseband I (in-phase)/Q (quadrature) and the INS error for performance analysis [20]. Several studies addressed the algorithms of the pre-filter and the centralized navigation filter [21,22]. Hwang, D.B., *et al*., provided a unified frame applicable to several different algorithms of GPS/INS deep fusion [23]. Xie, F., *et al*., proposed an adaptive robust deeply coupled GNSS/INS system based on a federated architecture which can process the GPS L1 and BeiDou B1 signals together [24]. Qin, F., *et al*., assessed the performance using low-cost IMU and vector tracking-based integration for high dynamic applications [25]. Recent work in [26] presented a federated GPS/INS deep coupling system employing three Kalman filters and providing adaptation ability to improve the navigation performance under various jamming conditions.

All of these reported aiding methods, however, inject the externally-estimated carrier Doppler directly into the tracking loop as a constant during the coherent integration period, which is at least 1 millisecond (ms). This limits the aiding rate not higher than 1000 Hz which is insufficient to the suppression of high dynamic stress errors. For example, the carrier Doppler may change up to 10.5 Hz over a 1 ms interval under a 2000 m/s^2^ acceleration. Therefore, the effects of the traditional aiding are restricted.

This paper proposes a sample-wise aiding method, which interpolates the aiding data onto each sampling point before controlling local reference signal generation to further eliminate the high-order dynamic errors with only minor modification of the receiver NCO (numerically controlled oscillator). Section 2 goes deep into the digital realization of the Doppler-aided PLL to explore the influence of the aiding data update rate on the scalar tracking performance. Theoretical derivation of the phase error transfer function reveals that the aiding effect has a lot to do with the product of the frequency of signal dynamics and the aiding update interval. Section 3 presents the sample-wise aiding method to break the performance limitation of the traditionally aided PLL, and simulates the effects of linear and spline interpolations in comparison with the traditional aiding. In Section 4 a testbed for the performance limitation of GPS/INS ultra-tight integration is implemented, which employs a digital IF (intermediate frequency) GPS signal simulator to test the tracking errors of a software GPS receiver ultra-tightly coupled with INS data in various high dynamic scenarios. The test results coincide with theoretical expectations and demonstrate that the sample-wise aiding can eliminate the dynamic errors much more effectively than the traditional aiding.

## 2. Performance of Traditional Doppler Aiding

Both the scalar tracking-based ultra-tight integration and the vector tracking-based deep integration use Doppler-aided tracking loops, which allows the INS or the integrated navigation filter to account for the platform dynamics such that the receiver can narrow its loop bandwidths. To simplify the analysis of the effects of the aiding data update rate, this section will focus on the dynamic process of the scalar tracking based PLL.

**Figure 1:**
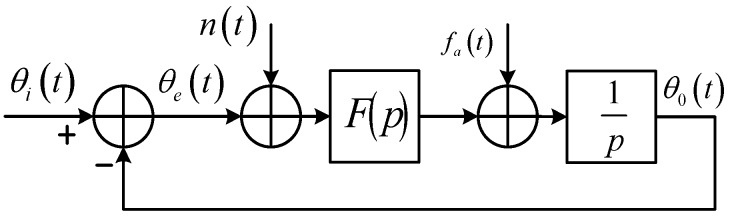
Linear phase model of Doppler-aided PLL.

According to the fundamental theory of PLL [27], the linear phase model of a PLL can be extended to describe the dynamic process of a PLL aided by external Doppler data, as shown in Figure 1.

In Figure 1, $\theta_{e}\left(t\right)=\theta_{i}\left(t\right)-\theta_{o}\left(t\right)$ is the phase error between the input phase $\theta_{i}\left(t\right)$ and the PLL output phase $\theta_{o}\left(t\right)$; $f_{a}\left(t\right)$ is the aiding Doppler provided by the INS estimate; F(p) denotes the transfer operator of the loop filter in the time-domain analysis, whose counterpart is F(jω) in the frequency domain, and F(s) in the Laplace domain.

For an unaided PLL, the closed-loop phase transfer function is $H\left(s\right)=\frac{F\left(s\right)}{s+F\left(s\right)}$, and the phase error transfer function is $H_{e}\left(s\right)=1-H\left(s\right)=\frac{s}{s+F\left(s\right)}$. The phase error $\theta_{e}\left(s\right)$ is determined by the input signal phase $\theta_{i}\left(s\right)$ and the phase noise n(s) as follows:

(1) $$\theta_{e}\left(s\right)=H_{e}\left(s\right)\theta_{i}\left(s\right)-H\left(s\right)n\left(s\right)$$

Generally the term “high dynamics” is embodied in two aspects: high frequency and large amplitude of the range/velocity/acceleration/jerk variations. For the case study of the behavior of the loop under high dynamics, a typical sinusoidal trajectory in the LOS (line of sight) direction is selected in this paper due to its following characteristics: (1) any complicated LOS dynamics can be decomposed into a number of sinusoidal components with different amplitudes and frequencies; and (2) it can provide severe high-order dynamics with continuous derivatives and limited range of motion, which is easy to control and convenient for testing. Therefore the LOS Doppler is simply modeled as:

(2) $$f_{d}\left(t\right)=A\sin\left(\omega t\right)$$

The carrier phase of the incoming signal is:

(3) $$\theta_{i}\left(t\right)=-\frac{A}{\omega }\cos\left(\omega t\right)+\theta_{0}$$

Since second-order and higher-order PLLs are not sensitive to frequency/phase steps, the steady state carrier phase tracking error is only caused by the first item in Equation (3):

(4) $$\theta_{e}(t)=-\frac{A}{\omega }\left|H_{e}\left(\omega \right)\right|\cos\left[\omega t+\Phi_{e}\left(\omega \right)\right]$$

It is seen that the sinusoidal dynamic stress error cannot be completely eliminated, and the amplitude of the error transfer function $\left|H_{e}\left(\omega \right)\right|$ represents the ratio of the amplitude of the phase error to that of the incoming phase.

For a Doppler aided PLL, it can be easily derived from Figure 1 such that:

(5) $$\theta_{e}\left(s\right)=H_{e}\left(s\right)\theta_{ia}\left(s\right)-H\left(s\right)n\left(s\right)$$

(6) $$\theta_{ia}\left(s\right)=\theta_{i}\left(s\right)-\theta_{a}\left(s\right)$$

(7) $$\theta_{a}\left(s\right)=\frac{1}{s}f_{a}\left(s\right)$$

where $\theta_{ia}\left(s\right)$ is defined as the equivalent input phase of the aided PLL, and $\theta_{a}\left(s\right)$ is the feedback phase introduced by Doppler aiding.

**Figure 2:**
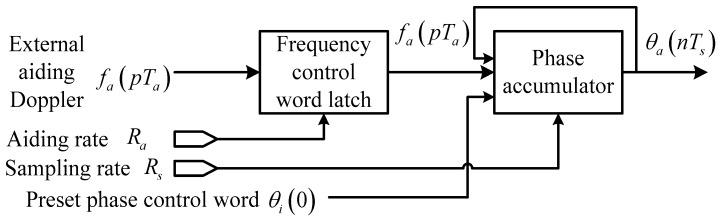
Feedback phase of traditionally aided DPLL.

Ideally, if the INS-estimated Doppler is exactly the derivative of the input phase, there will be $\theta_{ia}\left(t\right)=0$ and $\theta_{e}\left(s\right)=H\left(s\right)n\left(s\right)$, which means that the LOS dynamics can be completely eliminated by Doppler aiding. As a matter of fact, the signal frequency shift is also contributed by the receiver clock drift, the satellite clock drift, and even induced by the ionosphere/troposphere propagation, (which are beside the point of the paper). In this case, the design of the loop filter only needs to account for the phase noise instead of tracking the signal dynamics. The analog domain analysis, however, cannot cover all aspects of a digital realization. (1) In reality, a PLL in a modern receiver is implemented in its digital version—the DPLL where, traditionally, the aiding Doppler value remains constant over an external data update interval *T_a_* (which may be equal to, smaller than, or larger than the pre-detection integration time *T*; nevertheless most reported systems keep $T_{a}\geq T$ as considered in [19]); whereas the Doppler variation within this interval cannot be ignored in high dynamic situations; and (2) the carrier NCO control word actually comes from the Doppler estimate of the previous interval rather than the current interval. Taking these two factors into account, we replace the analog NCO integrator in Figure 1 with a digital accumulator operated at the sampling rate $R_{s}$, as shown in Figure 2. Note that another input of the adder before the integrator in Figure 1, namely the loop filter output, has been omitted in Figure 2, being dedicated to investigating the feedback phase of the aiding data.

Assuming that the aiding Doppler is provided at an update rate $R_{a}=1/T_{a}$, and the Doppler data update interval is the integral multiple of the digital signal sample interval $T_{s}$: $m=\frac{T_{a}}{T_{s}}=\frac{R_{s}}{R_{a}}$.

At the *n*th sample time $nT_{s}=\left(km+l\right)T_{s}$$=kT_{a}+lT_{s}$, l=0,1,⋯,m−1, the feedback phase can be expressed as $\theta_{a}(nT_{s})=\sum_{p=0}^{k-1}f_{a}(pT_{a})\cdot T_{a}+f_{a}(kT_{a})\cdot lT_{s}$. The equivalent input phase of the loop at each aiding Doppler update epoch (*n* = *km*, *l* = 0) is:

(8) $$\theta_{ia}(kT_{a})=\theta_{i}(kT_{a})-\theta_{a}(kT_{a})=\theta_{i}(kT_{a})-\sum_{p=0}^{k-1}f_{a}(pT_{a})\cdot T_{a}=\sum_{p=0}^{k-1}\left[\theta_{i}(pT_{a}+T_{a})-\theta_{i}(pT_{a})-f_{a}(pT_{a})\cdot T_{a}\right]+\theta_{i}(0)$$

Assuming that the INS can provide exact Doppler estimates synchronized with the LOS signal Doppler, we have $f_{a}\left(pT_{a}\right)=A\sin\left(\omega pT_{a}\right)$ for the sinusoidal input carrier phase in Equation (3). Substituting them into Equation (8) and neglecting the constant item (because it contributes nothing to the steady state tracking error), we obtain:

(9) $$\begin{array}{ll} \theta_{ia}(kT_{a}) & =\sum_{p=0}^{k-1}\left[-\frac{A}{\omega }\cos\left(\omega pT_{a}+\omega T_{a}\right)+\frac{A}{\omega }\cos\left(\omega pT_{a}\right)-A\sin\left(\omega pT_{a}\right)\cdot T_{a}\right]\\ & =A\sum_{p=0}^{k-1}\left[C_{a2}\left(\omega T_{a}\right)\sin\left(\omega pT_{a}\right)+C_{a1}\left(\omega T_{a}\right)\cos\left(\omega pT_{a}\right)\right]\cdot T_{a}\\ & =AC_{a}\left(\omega T_{a}\right)\sum_{p=0}^{k-1}\sin\left[\omega pT_{a}+\phi_{a}\left(\omega T_{a}\right)\right]\cdot T_{a} \end{array}$$

(10) $$\begin{array}{l} C_{a1}\left(\omega T_{a}\right)=\frac{1-\cos\left(\omega T_{a}\right)}{\omega T_{a}}=C_{a}\left(\omega T_{a}\right)\sin\left[\phi_{a}\left(\omega T_{a}\right)\right],\;C_{a2}\left(\omega T_{a}\right)=\frac{\sin\left(\omega T_{a}\right)-\omega T_{a}}{\omega T_{a}}=C_{a}\left(\omega T_{a}\right)\cos\left[\phi_{a}\left(\omega T_{a}\right)\right]\\ C_{a}\left(\omega T_{a}\right)=\sqrt{C_{a1}^{2}\left(\omega T_{a}\right)+C_{a2}^{2}\left(\omega T_{a}\right)},\;\phi_{a}\left(\omega T_{a}\right)=\mathrm{atan}2\left(1-\cos\omega T_{a},\sin\omega T_{a}-\omega T_{a}\right) \end{array}$$

When the product of the frequency of signal dynamics and the aiding update interval satisfies $\omega T_{a}\ll 2\pi$, the accumulation of discrete sinusoidal signals in Equation (9) can be approximated by the integration of the corresponding analog signal as the following:

(11) $$\theta_{ia}\left(kT_{a}\right)\approx AC_{a}\left(\omega T_{a}\right)\int_{0}^{kT_{a}}\sin\left[\omega t+\phi_{a}\left(\omega T_{a}\right)\right]dt=-\frac{A}{\omega }C_{a}\left(\omega T_{a}\right)\cos\left[\omega kT_{a}+\phi_{a}\left(\omega T_{a}\right)\right]+\frac{A}{\omega }C_{a}\left(\omega T_{a}\right)\cos\left[\phi_{a}\left(\omega T_{a}\right)\right]$$

Consequently, the phase error of the aided loop will follow the sinusoidal shape with the same angular frequency of the input signal phase as shown in Equation (4), yet with an amplitude of $C_{a}\left(\omega T_{a}\right)$ times that of the unaided PLL. The equivalent phase error transfer function of the aided loops is:

(12) $$H_{e-a}\left(\omega \right)=H_{e}\left(\omega \right)C_{a}\left(\omega T_{a}\right)\exp\left[\phi_{a}\left(\omega T_{a}\right)\right]$$

When $\omega T_{a}>2\pi$, we can find a frequency $\omega_{b}$ meeting $\omega_{b}T_{a}<2\pi$ and $\omega T_{a}=\omega_{b}T_{a}+m2\pi$, which yields $\theta_{ia}(kT_{a})\approx -\frac{A}{\omega }C_{a}\left(\omega T_{a}\right)\cos\left[\omega_{b}kT_{a}+\phi_{a}\left(\omega T_{a}\right)\right]+\frac{A}{\omega }C_{a}\left(\omega T_{a}\right)\cos\left[\phi_{a}\left(\omega T_{a}\right)\right]$. For this case Equation (12) still suffices. It is accurate enough, provided that the product of $\omega_{b}$ and $T_{a}$ satisfies $\omega_{b}T_{a}\ll 2\pi$, as can be validated by the simulation in Section 3.2.

**Figure 3:**
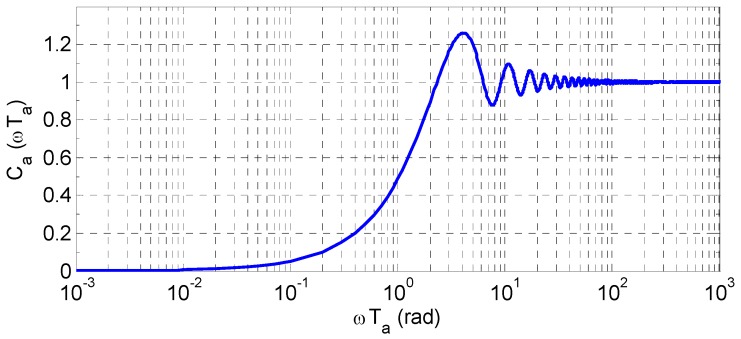
Ratio of phase error amplitudes of aided loop to unaided loop.

Thus, $C_{a}\left(\omega T_{a}\right)$ is the ratio of the phase error amplitudes of aided loop to unaided loop. Figure 3 shows how this ratio varies with $\omega T_{a}$ according to Equation (10). Note that the $C_{a}\left(\omega T_{a}\right)$ expression is applicable to any loop orders except for 1.

It can be seen that: (1) If $\omega T_{a}<2.3311$, the Doppler aiding can improve tracking performance by $C_{a}\left(\omega T_{a}\right)<1$; (2) When $\omega T_{a}\gg 2\pi$, $C_{a}\left(\omega T_{a}\right)\approx 1$, the aiding can hardly help to mitigate the dynamic stress error; (3) When $\omega T_{a}\ll 2\pi$, $C_{a}\left(\omega T_{a}\right)\approx \frac{1}{2}\omega T_{a}$, $\underset{\omega T_{a}\to 0}{\lim}C_{a}\left(\omega T_{a}\right)=0$. Hence, for the traditional Doppler aided loop, the aiding rate is expected to be as high as possible to efficiently reduce the dynamic stress error. However, the practical INS-offered data rate may not satisfy the cases with high frequency component or large amplitude of the LOS dynamics.

## 3. Sample-Wise Doppler-Aided PLL

### 3.1. Ideal Sample-Wise Doppler Aiding

**Figure 4:**
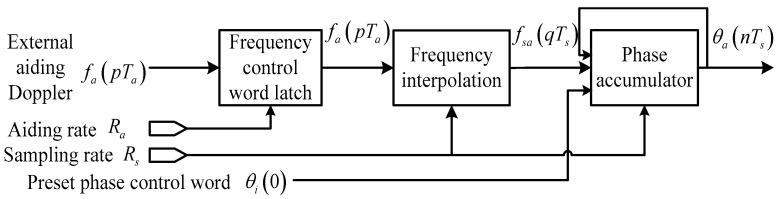
Feedback phase of sample-wisely aided DPLL.

In order to reduce the high dynamic stress error, it is effective to increase the aiding rate. Actually, the external aiding data can be interpolated to each sample point of the digital signal, and the control words of the carrier NCO for generating the local reference carrier can be updated at a rate up to the sampling rate $R_{s}$ without major modification of the receiver architecture, as shown in Figure 4. This is the main idea of the sample-wise Doppler aiding method proposed in this section. Compared with traditionally-aided DPLL in Figure 2, the only additional cost of sample-wisely aided DPLL is the frequency interpolation unit before the NCO phase accumulator.

In Figure 4 the control word of the carrier NCO is $f_{sa}\left(0:T_{s}:T_{p}\right)=\text{interp1}\left[f_{a}(0:T_{a}:T_{p})\right]$, where interp1[·] is the one-dimensional data interpolation function and $T_{p}$ is the time length of the data. In contrast to the traditionally aided DPLL, the equivalent input carrier phase in a sample-wisely Doppler aided DPLL becomes:

(13) $$\theta_{ia}(nT_{s})=\theta_{i}(nT_{s})-\theta_{a}(nT_{s})=\theta_{i}(nT_{s})-\sum_{q=0}^{n-1}f_{sa}(qT_{s})\cdot T_{s}$$

If there are still no aiding Doppler error and no time alignment error, ignoring the interpolation error (which will be investigated in the next subsection), we can substitute $f_{sa}\left(qT_{s}\right)=A\sin\left(qT_{s}\right)$ and $\theta_{i}(t)=-\frac{A}{\omega }\cos\left(\omega t\right)$ into Equation (13) to obtain:

(14) $$\theta_{ia}\left(nT_{s}\right)\approx -\frac{A}{\omega }C_{a}\left(\omega T_{s}\right)\cos\left[\omega nT_{s}+\phi_{a}\left(\omega T_{s}\right)\right]+\frac{A}{\omega }C_{a}\left(\omega T_{s}\right)\cos\left[\phi_{a}\left(\omega T_{s}\right)\right]$$

It is easy to meet that:

(15) $$C_{a}\left(\omega T_{s}\right)\approx \frac{1}{2}\omega T_{s}$$

Thereby the equivalent phase error transfer functions of the sample-wisely aided loop is:

(16) $$H_{e-sa}\left(\omega \right)=H_{e}\left(\omega \right)C_{a}\left(\omega T_{s}\right)\exp\left[\phi_{a}\left(\omega T_{s}\right)\right]$$

The equations indicate that the aiding performance can be significantly improved by increasing the aiding rate from $R_{a}$ to $R_{s}$. Smaller dynamic stress error can be achieved with a higher sampling frequency.

**Figure 5:**
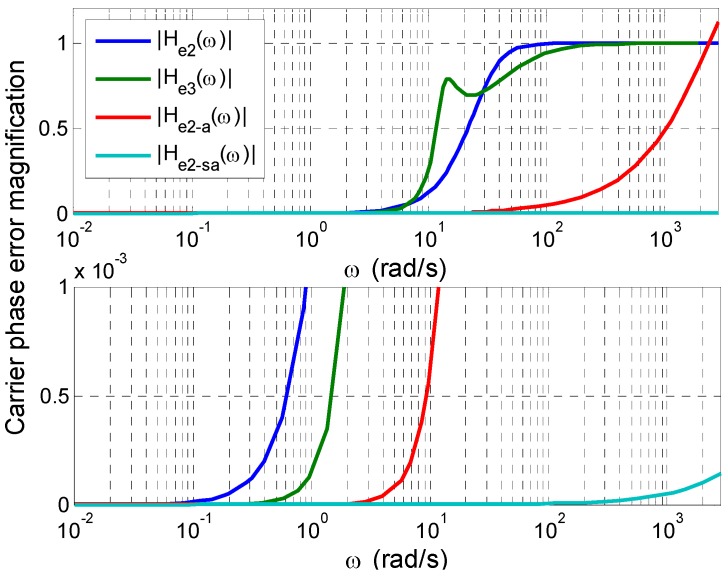
Amplitudes of phase error transfer functions.

As stated in [14], a second-order PLL is superior to a third-order PLL when aided. Taking typical third-order and second-order PLL designs in [2] as examples, Figure 5 plots the amplitudes of the phase error transfer functions of the unaided second-order PLL, the unaided third-order PLL, the traditionally-aided second-order PLL, and the sample-wisely-aided second-order PLL, respectively, with the PLL bandwidth *B*_PLL_ = 15 Hz, the Doppler aiding rate $R_{a}=1/T_{a}=1\mathrm{KHz}$, and the signal sampling rate $R_{s}=1/T_{s}=10\mathrm{MHz}$.

Figure 5 shows that:

1. When the frequency of the LOS dynamic vibration goes much higher than the natural frequency of the loop, the unaided carrier tracking loop has an amplitude magnification of near 1 with hardly any suppression on the dynamic stress error.
2. Except for the frequency range of 6.7 < *ω* < 28.6 (rad/s), the unaided third-order PLL shows better dynamic performance than the unaided second-order PLL.
3. For the dynamic vibrations with ω>2331rad/s, the traditional Doppler aiding cannot improve tracking performance efficiently.
4. Since $T_{s}$ is normally several orders of magnitude smaller than $T_{a}$, the phase error can be significantly reduced by sample-wise Doppler aiding.

### 3.2. Effects of Interpolation Techniques

In practice, we cannot obtain the exact value of the signal Doppler at each sample point by interpolation. This section explores the effects of different interpolation techniques on the dynamic stress error, still ignoring the errors in the INS estimates. Two typical interpolation approaches are investigated: (1) the linear interpolation; and (2) the cubic spline interpolation.

**Figure 6:**
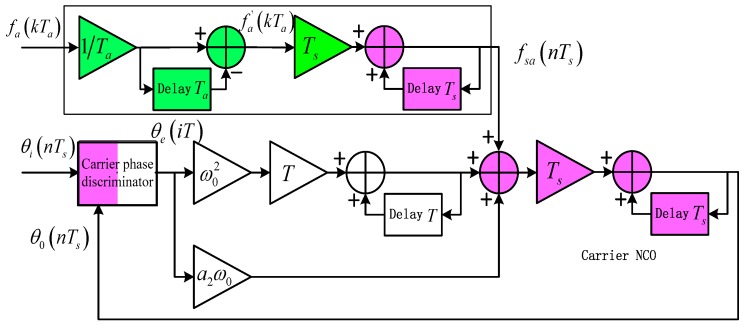
Block diagram of second-order PLL aided by linearly-interpolated Doppler.

A linear interpolation is generally the simplest that yields a good result when up-sampling. Figure 6 illustrates the block diagram of a second-order PLL sample-wisely-aided by the Doppler data linearly-interpolated from the INS data rate $R_{a}$ to the sampling rate $R_{s}$.

Figure 6 follows the phase model of Figure 1 in the time domain and omits the input noise for simplicity. The components shown in purple are operated at the sampling rate $R_{s}$ and often implemented in hardware for real-time processing; whereas the components shown in white and green are operated at the loop filter update rate R (usually the reciprocal of the pre-detection integration time T) and the INS data update rate $R_{a}$ respectively. One exception is the carrier phase discriminator, which is usually composed of the carrier lookup table, the multipliers for carrier and code wipe-offs, and the integration and dump accumulator. The accumulator performs low-pass filtering and down-samples the data from $R_{s}$ to R=1/T. The cost of replacing traditional aiding with sample-wise aiding is the linear interpolation unit shown in the top block of Figure 6, which can also be regarded as an extra stage of NCO accumulator.

In high dynamic situations, the simple linear interpolation may not meet the requirements of curve smoothness and approximation. More complicated up-sampling should be used to construct a smoother velocity and approximate complex trajectory. Among the interpolation techniques including polynomials, splines, Gaussian/low-pass processes, wavelets, *etc.*, the cubic spline is very popular for its tradeoff between accuracy and computational cost. The trajectory constructed by cubic splines makes the velocity, acceleration, and jerk (VAJ) continuous, and the VAJ of the initial and final positions can be flexibly configured [28,29].

**Figure 7:**
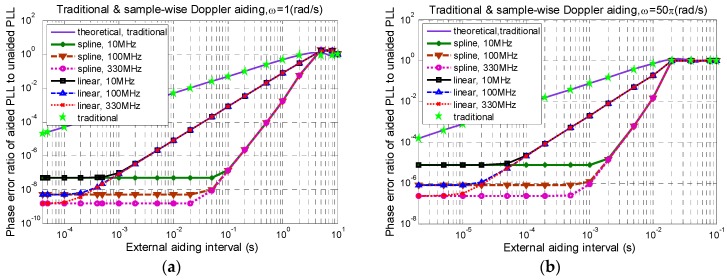
Ratio of phase error of aided PLL to unaided PLL, with (**a**) ω=1rad/s; (**b**) ω=50πrad/s.

Simulations are carried out for the two interpolation techniques from a wide range of INS data rates to three sampling rates. Figure 7 compared their effects on the ratios of phase error amplitudes of aided PLLs to unaided loops, with (a) ω=1rad/s, (b) ω=50πrad/s, respectively. The simulation results of traditional aiding are also included and compared with the theoretical prediction in Equation (10).

It can be seen that:

1. Equation (10) is accurate to quantify the effectiveness of traditional Doppler aiding, regardless of the aiding data error and the time alignment error.
2. When $\omega T_{a}>2.33\mathrm{rad}/s$, any aiding technique cannot suppress the dynamic stress error efficiently and, thus, is not worth the bother.
3. When *T_a_* begins to decrease from its upper threshold $T_{u}\left(\omega \right)\approx 2.33/\omega$, sample-wise aiding with linear interpolation shows better tracking performance than traditional aiding, yet its improvement cannot catch up with the spline interpolation, until that $T_{a}$ reaches a certain lower threshold, denoted by $T_{l}\left(\omega ,R_{s}\right)$. When $T_{a}<T_{l}\left(\omega ,R_{s}\right)$, the ratio of the phase error amplitudes of sample-wisely aided PLL to unaided PLL will be only determined by the theoretical limit $C_{a}\left(\omega T_{s}\right)\approx 0.5\;\omega T_{s}$, independent of $T_{a}$, and cannot be improved by employing other more advanced interpolation techniques. In addition, before reaching the lower threshold, there is a middle threshold $T_{m}\left(\omega ,R_{s}\right)$ of *T_a_*, such that for the $T_{m}\left(\omega ,R_{s}\right)<T_{a}<T_{u}\left(\omega \right)$ region, the improvement effect of sample-wisely aided PLL to unaided PLL is only determined by $T_{a}$ and the interpolation method, independent of $R_{s}$, and can be improved by employing other more advanced interpolation techniques.

It is interesting to divide each sample-wise aiding curve into the following four regions by using three thresholds of *T_a_* as shown in Table 1.

By contrast, there are only two regions for the traditional aiding: (1) $T_{a}<T_{u}\left(\omega \right)$: $C_{a}\left(\omega T_{a}\right)\to 0.5\;\omega T_{a}$; (2) $T_{a}\geq T_{u}\left(\omega \right)$:$C_{a}\left(\omega T_{a}\right)\to 1$.

It is also seen that the spline interpolation extends both the lower threshold $T_{l}\left(\omega ,R_{s}\right)$ and the middle threshold $T_{m}\left(\omega ,R_{s}\right)$ towards longer $T_{a}$ than the linear interpolation. Therefore, the pressure of high INS data updating rate can be relieved by employing advanced interpolation techniques to achieve the same performance as the linear interpolation. Additionally, higher dynamic frequency *ω* demands shorter $T_{a}$ thresholds. Yet the quantitative relationships of the $T_{a}$ thresholds $T_{l}\left(\omega ,R_{s}\right)$ and $T_{m}\left(\omega ,R_{s}\right)$ with the signal dynamic frequency *ω* and the sampling rate $R_{s}$ with different interpolation techniques are quite complicated and still under investigation.

## 4. Test Results and Discussion

### 4.1. Test Setups and Scenarios

The scalar tracking based GPS/INS ultra-tight integration has been implemented in our software receiver. Both the traditional Doppler aiding and the sample-wise aiding with spline interpolation are incorporated into the receiver PLL and DLL (delay locked loop), and their tracking performance in high dynamic scenarios can be tested by using our digital IF GPS signal simulator. The accurate INS Doppler estimates needed in the tests can simply be obtained from the user trajectory provided by the simulator and the satellite ephemerides.

Three factors have been considered in the design of dynamic scenarios for the tests: (1) to ensure that the receiver achieves steady state tracking before the aiding begins, no VAJ jumps are included in the trajectory, because carrier tracking loops are sensitive to dynamic jumps. For example, third-order loops are sensitive to jerk jumps; (2) the trajectory should be controlled within a limited spatial range; and (3) severe high-order dynamics should be included.

As a result the following sinusoidal user trajectory in the height direction is constructed:

$$h=h_{0}+D\left[1-\cos\left(\omega t\right)\right]$$

where, *h* is the height of the receiver antenna phase center, *h_0_* is the initial height, *D* is the amplitude of the sinusoidal trajectory, and ω is its angular frequency. In the following tests, we chose ω=1rad/s to simplify the control and calculation of dynamic parameters of different orders. For example, if *D* = 500 m, then the maximum velocity, acceleration, and jerk are 500 m/s, 500 m/s^2^, and 500 m/s^3^, respectively. With a satellite elevation angle of α=28.67°, the LOS velocity is:

(17) $$V_{LOS}=\left(D\sin\alpha \right)\omega \sin\left(\omega t\right)\approx 239.88\sin\left(t\right)$$

The receiver first tracks the high dynamic signals using a third-order PLL, and then changes to a second-order PLL aided by *a priori* known carrier Doppler after decoding the navigation message. Meanwhile the reception time (in GPS time) of each individual sample point can be obtained by using the single-SV (satellite vehicle) timing method proposed in [30]. For high dynamic scenarios, the known user trajectory rather than a static user location is utilized in the timing process. In this way the time alignment necessary for the aiding can be achieved.

The loop parameters recommended in Tables 5.6 in [2] are adopted in the tests. In addition, the PLL is configured with a loop bandwidth of 15 Hz and a loop update interval of 1ms, while the DLL taking a 1 Hz bandwidth and a 20 ms loop update interval. For traditional aiding, the external Doppler data are injected to the PLL every 1ms, and to the DLL every 20 ms whereas, for the sample-wise aiding, the receiver first interpolates the aiding data using cubic splines from 1000 Hz to the sampling rate, and then injects the interpolated carrier Doppler and code Doppler values into the carrier NCO and the code NCO, respectively.

Finally the code phase errors and carrier phase errors can be obtained by subtracting the simulator recorded data from the receiver measurements.

### 4.2. Aiding Effects on Carrier Phase Errors with no Thermal Noise in the Signal

**Figure 8:**
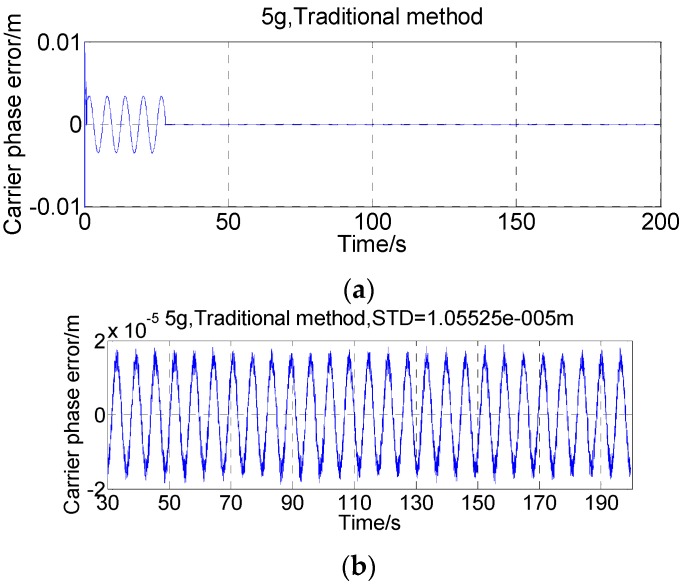
Carrier phase error using traditional aiding with 5 g dynamics and no thermal noise. (**a**) From unaided (0–30s) to aided (30–200s) status; (**b**) Aided period (30–200s).

To exclude the effects of thermal noises and cross-correlation interferences, a single-SV scenario is necessary to achieve high C/N_0_ (carrier-to-noise ratio) and, thus, high precision dedicated for the dynamic stress error test [30]. Although no noise is added to the output signal of the digital IF signal simulator, the C/N_0_ estimated by the receiver is around 128 dB-Hz owing to both the inevitable quantization noise and the limited range of the wideband-narrowband power ratio method (PRM) [31]. This is near the best PRM-estimated C/N_0_ achieved by our test platform so far. The carrier phase tracking errors in a 5 g (≈50 m/s^2^) dynamic condition are shown in Figure 8.

In the first 30 s, the receiver is in unaided state as the epoch time has not been solved. The top plot in Figure 8 shows that the carrier phase error is greatly reduced after external Doppler aiding being turned on. The bottom plot discards the first 30s data and examines the aided period. It can be seen that the carrier phase error remains a sinusoidal wave with the same angular frequency of 1 rad/s, as predicted by Section 2.

**Figure 9:**
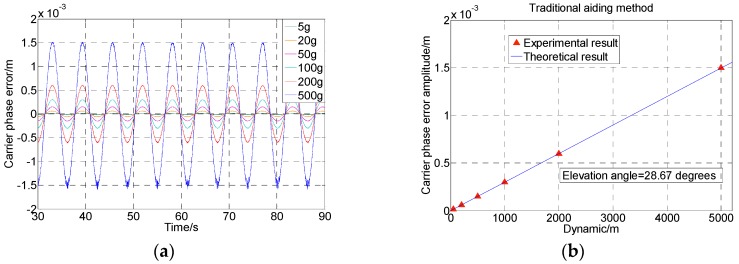
Carrier phase errors using traditional aiding with different dynamics and no thermal noise. (**a**) Test results; and (**b**) Comparison with theoretical error amplitude.

The tests have been conducted using six sets of signals with the dynamics of 5 g, 20 g, 50 g, 100 g, 200 g, and 500 g, respectively. The carrier phase tracking errors using traditional Doppler aiding are shown in Figure 9a. Figure 9b calculates their amplitudes by combining Equations (4), (10) and (12), and the experimental results shown in the red triangle symbols are well in accordance with the theoretical expectation in the blue line.

Table 2 summarizes the test results of the error amplitudes for the unaided third-order PLL and the aided second-order PLL and their ratios. The theoretical calculation of this ratio follows:

(18) $$r=\frac{\left|H_{e3}(j\omega )\right|}{C_{a}\left(\omega T_{a}\right)\left|H_{e2}(j\omega )\right|}\approx 230.04$$

The test results demonstrate the correctness of the equations of carrier tracking errors in Section 2. The small discrepancies between the test results and the theoretical value of the ratio are due to the impacts of the time alignment errors and the quantization errors of the test platform, as well as the round-off errors of the error amplitudes.

**Figure 10:**
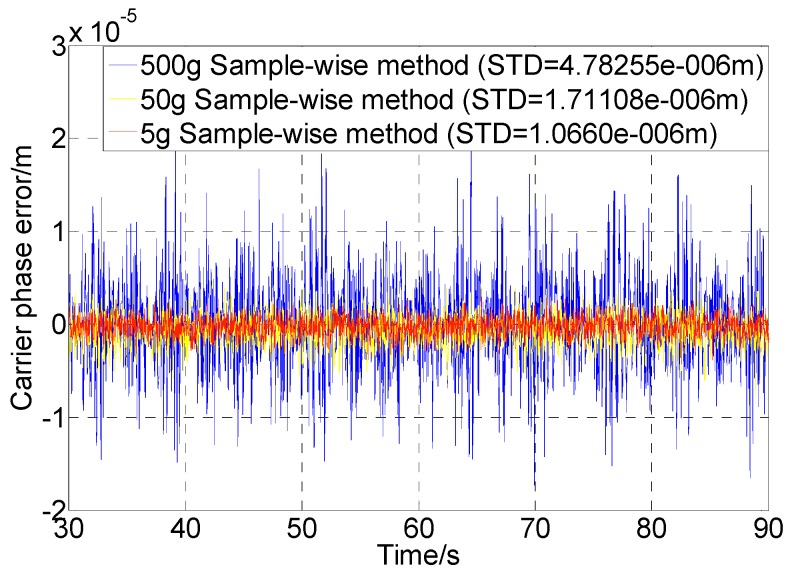
Carrier phase errors using sample-wise aiding with different dynamics and no thermal noise.

Corresponding to Figure 9a, Figure 10 shows the tracking results using the sample-wise aided PLL with 5 g, 50 g, and 500 g dynamics. It is seen that the dynamic errors have been further eliminated. It should be noted that the tracking error achieved by the test platform even in a static user scenario is at the 0.8 × 10^−6^ m level in terms of the standard deviation of the carrier phase error. The errors in Figure 10 are also contributed by the alignment errors between the simulator and the receiver (related to the signal dynamics) and the quantization errors.

### 4.3. Aiding Effects on Code/Carrier Phase Errors with a C/N_0_ of 80 dB-Hz

The carrier tracking loop is usually the weak link in a receiver and the carrier phase error characterizes the receiver dynamic performance. For the traditionally-aided receiver tracking in high dynamic conditions, however, the code tracking loop may also become the dynamically weak part since the DLL has a narrower bandwidth and a longer integration time and sometimes a narrow correlator spacing for the sake of noise and interference suppression. Both the carrier phase error and the code phase error are concerned in this subsection. The signal C/N_0_ is configured to be 80 dB-Hz in the test for the following two reasons: (1) the 1σ thermal noise code tracking jitter is about 0.0059 m under 80 dB-Hz and a narrow correlator spacing of 0.1chip. If the C/N_0_ is set too low, all the test results of the aided receiver will be buried in the thermal noise jitter; and (2) one job in the future is to experiment based on a RF (radio frequency) signal simulator and a RF signal collection device, where the 80 dB-Hz C/N_0_ has already been achieved at RF level using a single-SV scenario [31].

**Figure 11:**
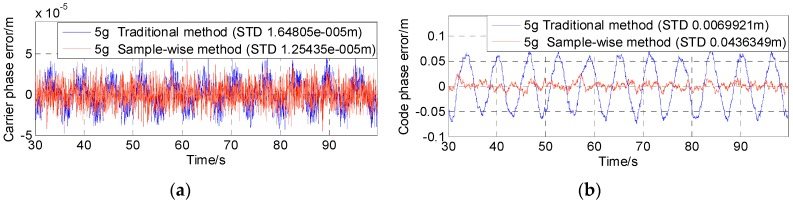
Tracking errors with 5 g dynamics and 80 dB-Hz C/N0. (**a**) Carrier phase error; (**b**) Code phase error.

**Figure 12:**
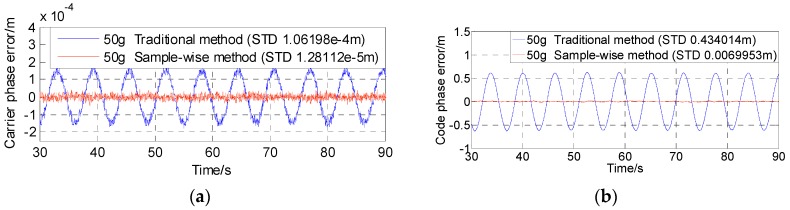
Tracking errors with 50 g dynamics and 80 dB-Hz C/N0. (**a**) Carrier phase error; (**b**) Code phase error.

**Figure 13:**
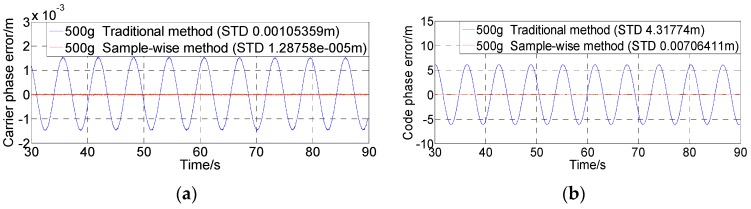
Tracking errors with 500 g dynamics and 80 dB-Hz C/N0. (**a**) Carrier phase error; (**b**) Code phase error.

**Figure 14:**
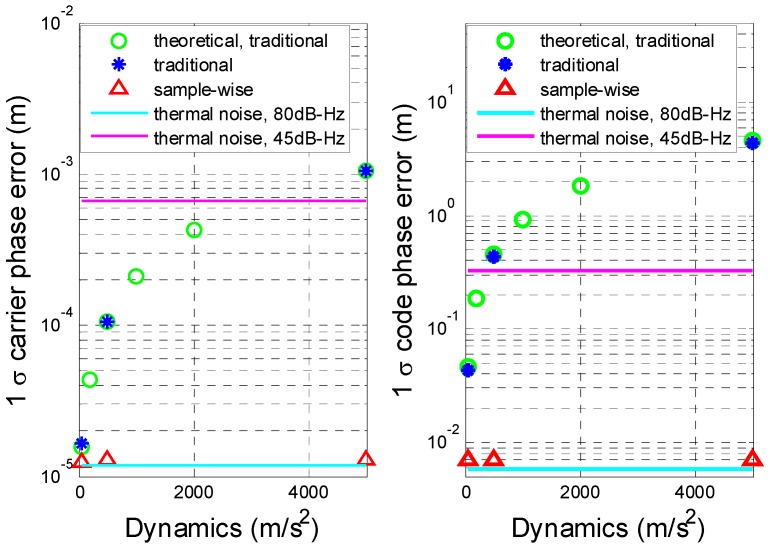
Comparison of tracking errors using traditional and sample-wise aiding.

Figure 11, Figure 12 and Figure 13 show the test results of the traditional aiding method and the sample-wise method with user dynamics of 5 g, 50 g and 500 g, respectively. Figure 14 summarizes the standard deviations of carrier tracking errors and code tracking errors of the two aiding methods in the test results. For comparison, Figure 14 also shows the theoretical values of the thermal noise floors at 80 dB-Hz and 45 dB-Hz (in normal signal conditions) as well as the theoretical values of the total 1σ errors at 80 dB-Hz (including contributions from both the dynamic stress and the thermal noise) for the traditional aiding method.

It can be seen that: (1) under a high C/N_0_ of 80 dB-Hz the dynamic stress error remains the main error source in all the traditional aiding results, and the total carrier phase errors becomes much closer to the test results in last subsection with the dynamics increasing; whereas all the sample-wise aiding results are dominated by the thermal noise jitters; and (2) although the dynamic stress errors can be significantly reduced by traditional Doppler aiding, they can still become dominant in carrier phase errors when the dynamics go higher than 3400 m/s^2^, and dominant in code phase errors when the dynamics go higher than 390 m/s^2^ under a normal C/N_0_ of 45 dB-Hz. As a contrast, the tracking errors remain almost the same values near the noise floor with the increase of user dynamics when using sample-wise aiding, which indicates that the dynamic stress errors have been almost completely eliminated by the sample-wise Doppler-aided receiver.

## 5. Conclusions

1. By examining the digital implementation of the Doppler-aided PLL in scalar tracking-based GPS/INS ultra-tight integration, a theoretical expression of the ratio of phase error amplitudes of an aided loop to an unaided loop is derived, which indicates how the aiding effects are impacted by the product of the frequency of signal dynamics and the INS aiding rate.
2. A new sample-wise aiding method in GPS/INS ultra-tight integration for high-dynamic, high-precision tracking has been presented. Its effectiveness and advantage over traditional aiding have not only been analytically expressed and numerically simulated, but also physically tested using a digital IF signal simulator and a software receiver.
3. The simulation with respect to the effects of two interpolation techniques on the sample-wise aiding performance helps to find the boundary conditions of the aiding methods in terms of the INS aiding data update rate.

Although the research in this paper is based on the scalar tracking architecture in GPS/INS integration, the sample-wise aiding method can be introduced into the vector tracking-based deep coupling to satisfy high-dynamic, high-precision requirements. In the vector tracking case, the navigation filter output can be interpolated to the sampling rate before controlling the local signal generation of each satellite channel. Similarly, the method can also be extended to the fields of map aiding and integration of GNSS with other wireless positioning. Related future work also includes the analysis of the performance limitation due to the INS data error and the misalignment between GPS and INS.

## Figures and Tables

**Table 1:** Four regions of sample-wise aiding in terms of *T_a_*.

Partitions of *T_a_*	Features of Aiding Effect
$T_{a}<T_{l}\left(\omega ,R_{s}\right)$	$C_{a}\left(\omega T_{s}\right)\approx 0.5\;\omega T_{s}$, $\omega T_{s}$ determined, independent of $T_{a}$ and interpolation techniques
$T_{l}\left(\omega ,R_{s}\right)\leq T_{a}<T_{m}\left(\omega ,R_{s}\right)$	decreasing as either $T_{a}$ or $T_{s}$ decreases
$T_{m}\left(\omega ,R_{s}\right)\leq T_{a}<T_{u}\left(\omega \right)$	$\omega T_{a}$ determined, independent of $T_{s}$, improved by advanced interpolation technique significantly (at kind of exponential rate)
$T_{a}\geq T_{u}\left(\omega \right)$	No much improvement even with smaller $T_{s}$ and advanced interpolation

**Table 2:** Test results of carrier phase errors using traditional Doppler aiding.

Dynamics (Acceleration)	Error Amplitude (m) for Unaided 3rd-Order PLL, B = 15 Hz	Error Amplitude (m) for Aided 2nd-Order PLL, B = 15 Hz	Ratio of Unaided to Aided Error Amplitudes
5 g	0.00345	1.50 × 10^−5^	230.00
20 g	0.0138	6.03 × 10^−5^	228.86
50 g	0.0345	1.49 × 10^−4^	231.54
100 g	N/A	3.00 × 10^−4^	N/A
200 g	N/A	6.00 × 10^−4^	N/A
500 g	N/A	1.50 × 10^−3^	N/A
